# Methicillin-Resistant *Staphylococcus aureus* in Pigs with Exudative Epidermitis

**DOI:** 10.3201/eid1309.061268

**Published:** 2007-09

**Authors:** Engeline van Duijkeren, Marc D. Jansen, S. Carolien Flemming, Han de Neeling, Jaap A. Wagenaar, Anky H.W. Schoormans, Arie van Nes, Ad C. Fluit

**Affiliations:** * Utrecht University, Utrecht, the Netherlands; † University Medical Center Utrecht, Utrecht, the Netherlands; ‡ National Institute for Public Health and the Environment, Bilthoven, the Netherlands

**Keywords:** MRSA, transmission, pig, human, PFGE, MLST, spa-typing, dispatch

## Abstract

Despite a strict control program for methicillin-resistant *Staphylococcus aureus* (MRSA) in human medicine in the Netherlands, MRSA was cultured from exudative epidermitis lesions of 4 piglets on a breeding farm, 20 pigs on a supplier farm, and 2 workers on these farms. The MRSA strains were indistinguishable, suggesting direct transmission.

Worldwide, methicillin-resistant *Staphylococcus aureus* (MRSA) causes hospital- and community-acquired infections in humans. In the Netherlands, the proportion of clinical human isolates that are methicillin resistant is still very low (2%) (*1*). A 2005 study in France identified pig farming as a risk factor for increased nasal colonization with *S. aureus* (*2*). The strains found in farmers were not found in nonfarmers but often caused swine infections, which suggests transmission between pigs and farmers. During 2004–2005, MRSA was cultured from 3 Dutch patients who had had contact with pigs (*3*). Investigators also found 6 carriers of MRSA among a group of 26 pig farmers. In the Netherlands, contact with pigs is now recognized as a risk factor for MRSA carriage.

## The Study

In June 2006, a Dutch farmer contacted the Pig Health Unit of the Veterinary Faculty of Utrecht University about an outbreak of exudative epidermitis among his swine. On his breeding farm (farm A), which had 200 sows (22.5 piglets/sow/year), a high preweaning mortality rate (20%) was caused by exudative epidermitis. Exudative epidermitis is a skin disease normally caused by *S. hyicus* and is usually an acute infection in suckling and weanling piglets. On farm A, a litter of 3-week-old piglets housed in a crate had clinical signs of exudative epidermitis. Other pigs on this farm had been unsuccessfully treated with ceftiofur, cefquinome, tylosin, and trimethoprim/sulfonamides.

A skin lesion sample from 1 piglet was sent to the Veterinary Microbiological Diagnostic Center of Utrecht University, where it was plated on sheep blood agar and MacConkey agar and incubated at 37°C for 24 h. No growth was seen on MacConkey agar, but large numbers of hemolytic white colonies were found on the sheep blood agar. These colonies were identified as *S. aureus* by colony morphology examination, Gram staining, catalase and coagulase testing, and API ID32 Staph (bioMérieux, Marcy-l’Etoile, France). No *S. hyicus* was found. Antimicrobial drug susceptibility was determined by using an agar diffusion method with IsoSensitest agar (CM471, Oxoid, Basingstoke, UK) and Neosensitab discs (Rosco, Taastrup, Denmark). The breakpoints used were those recommended by the Dutch Committee on Guidelines for Susceptibility Testing (*4*). The *S. aureus* was susceptible to enrofloxacin, trimethoprim/sulfamethoxazole, and fusidic acid and resistant to ampicillin, gentamicin, kanamycin, tetracycline, erythromycin, lincomycin, and tylosin. Because this *S. aureus* was resistant to multiple drugs, it was suspected of being MRSA, and presence of the *mec*A gene was confirmed by PCR (*5*).

Farm A was revisited 2 weeks later. Additional samples were taken from the skin lesions, nares, or both of 5 other 3-week-old piglets with exudative epidermitis from a different litter than the first piglet; from the nares of 1 healthy sow; and from the nares and throats of 2 veterinary students. The students had had contact with the pigs on the day the samples were taken and 1 week earlier. To investigate the source of the MRSA, samples were also taken from the nares of 12 healthy weanling pigs and 10 healthy gilts on a supplier farm (farm B) that had provided gilts for breeding to farm A. Nares samples were also taken from 2 farmers on farm B (Table). The samples were plated on sheep blood agar and incubated in tryptic soy broth, 4% saline, 1% mannitol, phenol red (16 μg/mL), ceftizoxime (5 μg/mL), and aztreonam (50 μg/mL). After incubation at 37°C for 48 h, broth cultures were plated on sheep blood agar and incubated at 37°C for 24 h. Suspected colonies were identified as MRSA, and antimicrobial drug susceptibility was determined as described above. MRSA was cultured from the nares of 1 student (farm A), 1 farmer (farm B), 1 sow and 4 piglets (farm A), 20 pigs (farm B), and from skin lesions of 3 piglets (farm A). Susceptibility testing showed that all MRSA isolates were susceptible to fusidic acid, trimethoprim/sulfamethoxazole, and enrofloxacin and resistant to ampicillin, tetracycline, gentamicin, and kanamycin. Of the 32 isolates, 18 were susceptible to lincomycin, tylosin, and erythromycin, and 14 were resistant to these antimicrobial drugs. Both resistance patterns were detected on both farms. The phenotypic resistance of the isolates to oxacillin was confirmed by Etest (AbBioDisk, Solna, Sweden) according to manufacturer’s guidelines. MICs were >128 mg/L for all MRSA isolates.

**Table:** Methicillin-resistant *Staphylococcus aureus* (MRSA) from 2 pig farms, the Netherlands, 2006

Sample source	No. investigated	Sample site (no.)	Farm	No. MRSA-positive pigs or persons*	MRSA-positive sample site (no.)	Resistance pattern†
Piglets	6	Skin (4) Nares (5)	A	6	Skin (4) and/or nares (4)	TKG or TKGTyLE
Sow	1	Nares (1)	A	1‡	Nares	TKG or TKGTyLE
Students	2	Nares (2), throat (2)	A	1	Nares	TKG
Farmers	2	Nares (2)	B	1	Nares	TKG
Gilts	10	Nares (10)	B	8	Nares	TKG or TKGTyLE
Weanlings	12	Nares (12)	B	12	Nares	TKG or TKGTyLE

*For some, >1 positive sample or 2 types of MRSA were obtained from the same pig or person. †T, tetracycline; K, kanamycin; G, gentamicin; Ty, tylosin; L, lincomycin; E, erythromycin; all isolates were also resistant to ampicillin. ‡2 isolates with different resistance patterns.

MRSA isolates were genotyped by using pulsed-field gel electrophoresis (PFGE) with *Sma*I according to the Harmony protocol (*6*), *spa* typing (*7*), and multilocus sequence typing (MLST) (*8*). Typing of the staphylococcal cassette chromosome (SCC*mec*) was performed by using PCR (*9*–*11*).

All isolates were nontypeable by PFGE with the *Sma*I restriction enzyme. Nontypeable MRSA associated with pig farming possesses DNA methylase, which methylates the *Sma*I-recognition sequence and leads to uninterpretable results (*12*). Genotyping showed that all isolates had *spa* type t011 and MLST 398 and SCC*mec* type IV (*ccrA/B* gene type 2, *mec* complex non–class A). Although 2 distinct resistance profiles were observed, all isolates belonged to the MLST/*spa* genotype associated with pigs in the Netherlands.

## Conclusions

Because farm B regularly sells gilts to farm A, farm B is probably the source of the MRSA isolated from farm A. Farm B is a closed farm that has not purchased pigs since 1996. Further research is necessary to identify the source of MRSA on farm B.

Since 2002, human MRSA isolates sent to the National Institute for Public Health and the Environment by the regional laboratories are typed by PFGE, and nontypeable MRSA in persons who are not in contact with pigs is rare. The MRSA-positive farmer and student had no other known risk factors for MRSA carriage. The differences in the resistance patterns may be caused either by erythromycin-lincomycin-tylosin resistance genes located on mobile elements such as plasmids or transposons (like Tn*554*) or by differences in the expression of the resistance genes.

Transmission of MRSA between pigs and pig farmers has been previously reported by Voss et al. (*3*). However, to our knowledge, ours is the first report of culturing MRSA from clinically diseased pigs. The infected piglets were only 3 weeks of age, which suggests that they might have been infected through contact with their mother. The isolation of MRSA from piglets with exudative epidermitis was unexpected. That large numbers of *S. aureus* but no *S. hyicus* were cultured from the skin lesions indicates clinical relevance.

The sale of pigs to many different breeding farms favors the spread of MRSA. It was recently reported that 209 (39%) of 540 finishing pigs at Dutch slaughterhouses were MRSA positive and that all MRSA had MLST 398 and were resistant to tetracycline (*13*). Antimicrobial drugs, especially β-lactams and tetracyclines, may select for this MRSA strain. Farm A used many different antimicrobial drugs, including third-generation and fourth-generation cephalosporins, for treatment of exudative epidermitis; farm B regularly used amoxicillin. The diseased piglets were treated with enrofloxacin and recovered. Healthy pigs carrying MRSA should not be treated with antimicrobial drugs. Currently, no precautions are taken before and during slaughter of MRSA-positive pigs.

In conclusion, MRSA was cultured from clinically diseased and asymptomatic pigs. Colonization with MRSA seems to be widespread in Dutch pigs; supplier farms that sell MRSA-colonized pigs to other farms play a role in spreading the organism. Because the Netherlands exports pigs to other countries, further research on the prevalence of MRSA in pigs in foreign countries is warranted.
